# Preferences for services in a patient’s first six months on antiretroviral therapy for HIV in South Africa and Zambia (PREFER): research protocol for a prospective observational cohort study

**DOI:** 10.12688/gatesopenres.14682.2

**Published:** 2024-01-09

**Authors:** Mhairi Maskew, Vinolia Ntjikelane, Allison Juntunen, Nancy Scott, Mariet Benade, Linda Sande, Pamfred Hasweeka, Prudence Haimbe, Priscilla Lumano-Mulenga, Hilda Shakewelele, Mpande Mukumbwa-Mwenechanya, Sydney Rosen

**Affiliations:** 1 Health Economics and Epidemiology Research Office, Faculty of Health Sciences, University of the Witwatersrand Johannesburg, Johannesburg, Gauteng, South Africa; 2 Global Health, Boston University, Boston, MA, 02118, USA; 3 Department of Medical Microbiology, Amsterdam University Medical Center, Amstersdam, The Netherlands; 4 CHAI-Zambia, Clinton Health Access Initiative, Lusaka, Zambia; 5 MOH Zambia, Ministry of Health, Lusaka, Zambia; 6 Center for Infectious Disease Research in Zambia, Lusaka, Lusaka Province, Zambia

**Keywords:** HIV, South Africa, Zambia, antiretroviral therapy, retention, models of care

## Abstract

**Background:**

For patients on HIV treatment in sub-Saharan Africa, the highest risk for loss from care remains the first six months after antiretroviral (ART) initiation, when patients are not yet eligible for differentiated service delivery (DSD) models that offer lower-burden, patient-centred care and thus improve treatment outcomes. To reduce early disengagement from care, the PREFER study will use a sequential mixed-methods approach to describe the characteristics, needs, concerns, and preferences of patients in South Africa and Zambia 0-6 months after ART initiation or re-initiation.

**Protocol:**

PREFER is an observational, prospective cohort study of adults on ART for ≤6 months at 12 public healthcare facilities in Zambia and 18 in South Africa. Its objective is to describe and understand the needs and preferences of initiating and re-initiating ART clients to inform the design of DSD models for the early HIV treatment period, improve early treatment outcomes, and distinguish the barriers encountered by naïve patients from those facing re-initiators. It has four components: 1) survey of clients 0-6 months after ART initiation (identify characteristics and preferences of clients starting ART); 2) follow up through routinely collected medical records for <24 months after enrollment (describe resource utilization and patterns and predictors of engagement in care); 3) focus group discussions and discrete choice experiment (explore reported barriers to and facilitators of retention); and 4) in South Africa only, collection of blood samples (assess the prevalence of ARV metabolites indicating prior ART use).

**Conclusions:**

PREFER aims to understand why the early treatment period is so challenging and how service delivery can be amended to address the obstacles that lead to early disengagement from care. It will generate information about client characteristics and preferences to help respond to patients’ needs and design better strategies for service delivery and improve resource allocation going forward.

## Introduction

Rapid, same-day, and community-based initiation of antiretroviral therapy (ART) for HIV has shifted the challenge of achieving optimal outcomes in HIV treatment onto retention in care after a patient has started ART. The highest risk for loss from care consistently remains a patient’s first six months after ART initiation ^1^. Dubbed the “early retention” period ^2^, this interval accounts for roughly three quarters of all first-year attrition from HIV programs in sub-Saharan Africa ^3^. In South Africa, 26% of patients were lost to follow up by 6 months after initiation in a recent trial of a case management intervention ^4^, and attrition was 35.6% by 6 months in a recent observational study ^5^.

Patient characteristics and service delivery characteristics both contribute to high attrition during the early treatment period ^1^. After the World Health Organization (WHO) began recommending universal treatment access in 2016, the median CD4 count of new ART initiates rose substantially, reflecting the much higher proportion of asymptomatic, “healthy” patients than in the past ^6^, despite a fairly consistent minority of a quarter to a third continuing to present with very low CD4 counts ^7, 8^. The proportion of patients who are re-initiating ART after previously interrupting care is also climbing. A recent modeling exercise estimated that in 2020, fully 58% of those who tested positive for HIV were already aware of their positive status ^9^ and thus may have declined an earlier opportunity to start or remain on treatment. Two small studies in South Africa identified ARV metabolites—evidence of recent exposure to ARV medications—in 53% and 19% of self-reported naïve ART initiators in Limpopo and KZN provinces, respectively ^10, 11^. Individuals who know their HIV-positive status but are not on ART likely face multiple barriers to starting treatment; those who both know their status and have already started and stopped ART at least once (re-initiators) may require additional support to remain in care once they re-initiate care.

The service delivery landscape has transformed at many points in the HIV treatment cascade in recent years, most notably through the introduction of rapid and same-day ART initiation for those not yet on treatment ^12^ and the development of patient-centred differentiated service delivery (DSD) models for those already established on ART for at least 6 months ^13^. Neither same-day initiation nor most current DSD models, however, offer solutions for patients during the first six months after initiation ^14^. The model of care for the early treatment period that is offered to most newly-initiating and re-initiating patients has evolved little from its original outlines, though COVID-19 restrictions precipitated some reductions in required numbers of clinic visits and increases in dispensing intervals ^15^.

A first step in designing new models of care for the early treatment period is to gain a comprehensive understanding of patients’ needs, concerns, resources, and preferences for service delivery during this period. Treatment and retention in the first 6 months after ART initiation could be improved if patients were triaged to receive more or less support based on a combination of known risks to retention in care and patient preferences for how little or how much interaction, and what kinds of interaction, with the health system are desired.

In the PREFER study, we will use a sequential mixed-methods approach to survey a sample of patients in South Africa and Zambia at various points between months 0 and 6 after ART initiation to develop a detailed profile of different groups of patients who may be best served by different models of care. The specific objectives of the study include describing the characteristics and preferences of new and re-initiating ART clients, exploring their concerns in depth through focus groups, identifying associations between client characteristics and early treatment outcomes, and assessing differences between naïve and non-naïve treatment initiators.

## Protocol

### Overview

**Figure 1.**
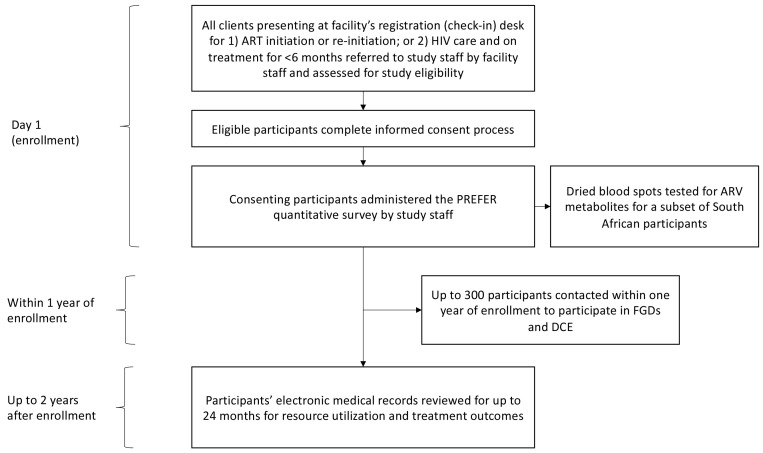
Components and flow of the PREFER study.

PREFER is an observational, prospective cohort study in South Africa and Zambia that aims to inform the design of service delivery models for the early HIV treatment period. Its primary objective is to describe and understand the needs of initiating and re-initiating ART clients in order to inform the design of DSD models for the early HIV treatment period and improve early treatment outcomes. It has four components: 1) a survey of clients during the period from 0 to 6 months after ART initiation; 2) follow up through routinely collected medical records for a maximum of 12 or 24 months after study enrollment, depending on the study country; 3) focus group discussions (FGDs) and a discrete choice experiment (DCE) to explore specific issues raised in the survey; and 4) in South Africa only, collection of blood samples from a subsample of who self-report that they are not currently on ART and are initiating ART on the day of study enrollment to assess the prevalence of ARV metabolites indicating prior ART use ( Figure 1).

PREFER is registered at Clinicaltrials.gov as NCT05454839 (South Africa) and NCT05454852 (Zambia).

### Study sites

PREFER will be conducted at 18 healthcare facilities in South Africa and 12 in Zambia ( Table 1). Study sites represent the facilities participating in the SENTINEL study of the AMBIT Project ^16^. For SENTINEL, local study teams first identified provinces and districts that were accessible, had a high burden of HIV, utilized the national electronic medical record system, and jointly provided diversity in setting (rural, urban), facility size, and nongovernmental support partners. Within each district, SENTINEL then selected a set of facilities that represented the desired diversity and were large enough to provide the sample sizes required. These were chosen with engagement from the relevant Departments and Ministries of Health, including national and district government health officials with responsibility for the sites. Further information about SENTINEL and preliminary results can be found at www.sites.bu.edu/ambit.

**Table 1.** PREFER study sites.

Facility	Setting	ART patients as of August, 2021	Average ART initiates per month, 2021
**South Africa**			
*West Rand District (Gauteng Province)*			
Clinic	Urban	1,783	16
Clinic	Rural	1,803	17
Clinic	Urban	1,897	16
Clinic	Urban	2,116	37
Clinic	Rural	2,301	13
Clinic	Urban	2,959	30
*Ehlanzeni District (Mpumalanga Province)*			
Clinic	Urban	6,365	49
Clinic	Rural	3,553	24
Clinic	Rural	1,946	9
Clinic	Rural	3,001	28
Community Health Centre	Urban	5,234	105
Health Centre	Urban	5,523	43
*King Cetshwayo District (KZN Province)*			
Clinic	Rural	1,182	11
Clinic	Rural	1,509	11
Clinic	Rural	2,231	10
Clinic	Rural	3,361	23
Clinic	Rural	5,190	34
Clinic	Urban	7,934	34
**Zambia**			
*Lusaka Province*			
Urban Health Centre	Urban	2,370	21
Referral Rural Health Centre	Rural	4,060	35
Health Center	Peri-urban	3,754	23
First Level Hospital	Urban	10,794	131
First Level Hospital	Urban	13,386	121
First Level Hospital	Urban	6,954	45
*Central Province*			
District Hospital	Rural	3,397	16
Urban Clinic	Urban	6,805	48
Urban Health Centre	Urban	3,753	17
Urban Health Centre	Urban	2,855	21
Mission Hospital	Rural	2,397	17
District Hospital	Rural	4,326	18

### Study population, informed consent, and enrollment

We will sequentially recruit adult (≥18 years old) ART patients on treatment for ≤6 months who present at the study sites for ART initiation, routine care, or unscheduled HIV-related care and provide written informed consent. We will exclude anyone who is unable to communicate in any of the languages into which the survey has been translated, is unwilling to take the time required to complete the survey on the day of consent, or who, in the opinion of study staff, is physically, mentally, or emotionally unable to participate in the study. Eligibility will be determined through completion of a survey screening form, which will also allow us to compare the sex and age distribution of the population enrolled in the survey with those of the full potentially eligible population (Extended data - Supplementary file 1).

At the study sites, clinic staff will inform potentially eligible patients that they may be eligible to participate in a research study when the patient checks in at the reception desk (i.e. as they enter the facility and make their initial contact with facility staff). Patients will be recruited consecutively as they arrive at the facility, based on availability of study interviewers. We expect this process to generate a representative sample of patients over the course of each enrollment day, accurately reflecting the diversity of eligible potential participants.

Written informed consent will be sought from all eligible participants, covering the survey, medical record review, agreement to be contacted for later focus group participation, and agreement to blood sample testing for prior ARV exposure (Extended data - Supplementary file 2). Participants will be informed of the risks or discomforts that may come from study participation, their rights as participants, data security and confidentiality, and information to contact the study team if questions or concerns arise. The consent process and questionnaire will be administered in a confidential space by a trained research assistant in a private location at the clinic during the periods while patients are waiting in queues for facility services (consultations or medication pickups). Participants will be assured that they will not lose their places in the queue as a result of study participation. Patients who start the survey but do not have time to complete it before reaching the front of the queue will be asked to return to the research assistant after receiving services, in order to complete the survey. We anticipate that each interview will last 75 minutes, including the consent process. Participants will be offered light refreshments during the study and will be given a token valued at approximately US$10 to thank them for their time and participation.

### PREFER survey of client characteristics and preferences

The PREFER survey instrument aims to describe the characteristics, experiences, concerns, and preferences of clients initiating or re-initiating ART. It will be a structured questionnaire designed for primarily quantitative analysis but with some open-ended questions (Extended data - Supplementary file 3). Questions will build on previous work of the AMBIT Project and on the authors’ experience in studying retention in HIV care ^1, 3, 17– 19^. The questionnaire has eight substantive sections, addressing the respondent’s demographics and socio-economic status, HIV testing history, HIV treatment history, current HIV care and treatment experience, other healthcare, preferences for features of treatment delivery, expectations, and costs of seeking care. Questions are designed to elicit information about participants’ past experiences with the healthcare system and identify changes that would potentially improve their future experiences.

As PREFER is a descriptive study that aims to describe participants’ self-reported experiences and preferences, rather than comparing outcomes or testing a hypothesis, sample sizes were chosen to optimize the use of study resources and time availability. Using the expected number of eligible patients at each study site, the number of such patients who are expected to visit the sites during the data collection period, and an anticipated data collection period of 90 days in each country, the study protocols allow a maximum enrollment of 2,500 participants per country. We anticipate that actual enrollment will be somewhat less than this, with a minimum target of an average of 50 participants per study site, or 900 in South Africa and 600 in Zambia; we expect these sample sizes to be sufficient to generate sufficiently precise results to the survey’s quantitative questions.

### Medical record review for outcomes and resource utilization

Using identifiers collected as part of the structured questionnaire, we will also collect follow up data from routine medical records for the period from each participant’s initial data entry (first presentation for testing or care) to 12 months after study enrollment, with an additional round of medical record data collection up to 24 months after study enrollment in South Africa. The objective of this component of the study is to describe early treatment outcomes and, using data from the survey, identify associations between client characteristics and their outcomes, in order to better match early treatment interventions with client needs.

Medical record data will be drawn from Tier.Net in South Africa and Smartcare in Zambia, paper records and registers maintained at the study sites, and other databases, such as South Africa’s National Health Laboratory Services database, to ascertain whether patients are retained in care during the first 6 months on ART. We will collect data on HIV treatment history and current engagement, medications dispensed, laboratory tests performed, comorbidities, and other healthcare provided by the site and recorded in the EMR during the study period. We note that the EMRs in both countries are largely limited to HIV care; they may capture data on tuberculosis and other conditions but generally do not contain information about services provided by other departments within the clinics.

### Post-enrollment focus group discussions to explore clients’ concerns and preferences

Participants will be asked during the consent process for their agreement to be contacted by telephone or e-mail at any time during the 12 months following study enrollment and invited to participate in a FGD. Focus groups will address the concerns raised by survey participants to explore in-depth the challenges clients face in the early treatment period.

We anticipate inviting up to 300 participants to participate in FGDs of up to 10 participants in each of up to 30 groups, 15 per country. We will purposively select PREFER sites which have sufficient numbers of enrollees and represent a diversity of settings and purposively identify participants who have a record of missed visits or who have expressed concerns about their care in the quantitative survey. Patients will be contacted by telephone or email, depending on their preference stated at enrollment. Study staff conversant in English and the appropriate local language and trained in qualitative research methods and human subjects protection will conduct the FGDs.

Before commencing the FGD, all participants will be briefly screened for eligibility and study staff will proceed with the informed consent process. We will document informed consent with a signature or a thumb-print and capture basic demographic information for each participant to allow linkage to their survey results. FGD leaders, who will be trained study research assistants, will remind all participants that anything said during the discussion must remain private and should not be shared beyond the group. The FGDs will conducted in a private setting in the clinic or community.

To guide the discussion and probe for more in-depth responses, FGD leaders will use an FGD guide developed based on responses to the PREFER survey and focusing on specific issues or concerns that participants raise in the survey (Extended data - Supplementary file 4). Anticipated topics, which may vary by country, include HIV treatment experience, patient preferences for receiving HIV treatment, and patient expectations of HIV care. The FGD will be audio recorded and the leaders will also take notes during the interview. FGDs should take no longer than two hours.

Prior to starting the focus group discussions, participants will also be asked to participate in a brief discrete choice experiment (DCE) to rank their specific preferences for features of treatment delivery, such as visit frequency and medication refill duration and location, in the first six months of care. The DCE will provide a series of hypothetical scenarios for receiving ART, each of which contains a combination of attributes that emerge as important in the survey, which we expect to include location, waiting time, months of ART dispensed at a time, friendliness of providers, cost, and/or other attributes. The DCE instrument will include a maximum of 10 attributes with no more than three response levels each. Each participant will evaluate a maximum of nine choice sets—two scenarios with a different hypothetical combination of attributes—with one repeated to check for internal consistency (Extended data - Supplementary file 5). Participants will select their preferred option, then be asked if they would use that option if it were available. We expect the DCE to take approximately 15 minutes per participant and to be administered in a quiet, private space before the FGD commences. DCE results will be combined with FGD outcomes to develop a more nuanced description of clients’ needs and preferences.

### Biological sample collection at South African sites to estimate prior ART exposure

For a subsample of South African study participants who are enrolled on the day of ART initiation or re-initiation who self-report as ART-naïve or not having been on treatment for a least 90 days, a dried blood specimen (DBS) will be collected from PREFER survey participants at some study sites. The objective of this component of the study is to add to the evidence base about the proportion and characteristics of the large group of clients who have prior ART experience but do not reveal this to healthcare providers. As these clients have, by definition, previously faced barriers to ART retention, understanding more about them is essential to improving early treatment outcomes.

Sites were chosen based on willingness of providers to create the dried blood spots after obtaining venous blood sample for routine ART initiation blood tests. Blood is drawn routinely as part of the standard of care ART initiation process in South Africa. DBS specimens will be extracted from the routinely collected blood samples drawn by existing clinic staff (nurses or phlebotomists), who are trained in safe and sterile collection techniques. For the study, a sample of 50μml will be extracted from an existing EDTA vacutainer tube with a capillary tube and placed onto a Whatman Protein 903 filter paper. A minimum of 3 dried blood spots will be completed for each patient. Specimens will be allowed to air dry for 2 hours after being taken, or according to assay instructions. Specimens will be covered with glycine weighing paper after drying. Collected, dried specimens will be stored in a -4°C freezer and be shipped in 6-weekly batches via courier to the University of Cape Town Division of Pharmacology, PK Laboratory for analysis. Specimens will be batch-screened for the presence of tenofovir diphosphate.

Participants who meet the inclusion criteria for this subsample (initiating or re-initiating ART and self-reporting as ART-naïve or not having been on treatment for HIV for at least 90 days) will be consecutively enrolled up to a maximum of 200 participants from all participating study sites combined. Enrollment in this subsample will begin when the consumables required for DBS creation have been procured and staff have been trained on standard operating procedures for creating a DBS. Consent for biological sample collection is included in the overall survey consent form.

### Data management

A screening register will be kept by the study research assistants to record the consent process and keep track of those who do not consent, to allow us to determine if our sample is biased by patient characteristics due to differential consent. The screening register will not contain any individual identifiers. It will request age category, gender, and months on ART, as needed to determine survey eligibility only.

Patient survey responses will be entered live at the time of the interview into an electronic database on Survey CTO (Dobility 2023) using handheld tablets. If there are power failures, data will be entered onto paper study forms and then transcribed into a database at the local study office. Survey data will be converted to SAS, STATA, or R for final cleaning and data analysis. All analytic databases will be password protected with access restricted to the members of the study team. All survey respondents will be assigned a seven-digit, sequential identification number. The study ID number will be used to identify individual subjects in the study databases and to link survey response data to patient retention outcomes data and for all data analysis. Once data are linked, identifiers will be removed from the analysis file and all subjects will be assigned a random study ID, which will be used for all analysis.

FGD audio files will be transcribed verbatim, then transcribed and translated into English for analysis.

Data for this study will be accessible only by key study personnel. Study ID numbers will be used in place of names or identifying information to safeguard participants’ privacy. All physical consent forms, questionnaires, and notes will be stored in locked cabinets, with access restricted to study personnel. All data will be collected on encrypted devices and stored on secure drives, with access restricted to study personnel. A fully anonymized data set may be posted to a public data repository when the protocol has been closed, if required by the funder and journal. 

### Data analysis and dissemination

We will first create a descriptive summary of the demographic and clinical characteristics of participants enrolled in the study and responses to each question using frequencies and simple proportions for categorical variables and medians with interquartile ranges for continuous variables. We will then stratify these baseline survey responses by site characteristics, time on ART, self-reported naïve v non-naïve treatment status, and/or patient characteristics such as age and sex, as data allow. Data will not be pooled across countries, but differences in results by country will be noted and discussed.

Utilizing follow up data collected from patient medical records, we will conduct a crude analysis reporting simple proportions with 95% confidence intervals of patients disengaging from care, defined as missing a scheduled clinical or medication pickup visit during the first 6 months after treatment initiation by more than 28 days. Next, we will compare the proportions of patient disengaged from care by key variables including age, gender, clinical stage at ART initiation, site characteristics, time on ART, and naïve v non-naïve treatment status.

If enrollment numbers allow, the analysis will include a simple unadjusted comparison of outcome groups (retained or disengaged) with respect to baseline predictors of outcomes. Potential predictors include demographic and clinical variables and geographic and facility-level factors (urban vs rural setting, facility type). Using a log-linear regression model, we will next estimate crude risk ratios and crude risk differences and their corresponding 95% confidence intervals. If any important differences are observed, we will proceed with an adjusted model. Should crude stratification techniques reveal potential effect measure modification, these analyses will not be adjusted but rather reported as stratified output.

For the FGD output, we will conduct a content analysis of the qualitative data using the Framework Method ^20^. We will use a combination inductive-deductive approach wherein most codes will be identified *a priori*, aligned with the FGD guide and literature, but the coding process will allow for additional codes to emerge to form a final analytical framework. Data will be charted, summarized, and quotations will be presented to illustrate key points when appropriate. Results will be presented stratified by country and urbanicity of sites.

To analyze the DCE survey data collected before the FGDs, we will estimate the impact of each attribute on a participant’s choice using a conditional logit model and a random effects probit model. The DCE results will be triangulated with the survey and FGD finding to complement PREFER overall conclusions about client preferences.

For the analysis of prior ART exposure, the presence of TDF above 0.02μg/ml in blood samples will be considered as positive for previous ART use in the previous 3 months or as advised by the laboratory. We will report the proportion of those who have evidence of usage of TDF and, where possible, use the quantified measure to estimate frequency of ART usage, in the preceding 90 days. If sample size allows, we will also identify client characteristics associated with prior ART exposure, using survey responses and medical records.

The study results will be disseminated in several ways. The primary audiences for the results of this study are the South African National Department of Health and Zambian Ministry of Health and their partners, who will use the findings to improve retention in care during the early treatment period. In addition, we will use the results to inform the design of one or more new models of service delivery, which we expect to implement and evaluate during a later stage of this project. Many of the findings will also likely be of broader interest in South Africa and other countries and will be made as widely available as possible, through journals, websites, and conferences. Only aggregated, stratified data will be presented; it will not be possible to identify any individual patients from any of the results that are presented.

### Limitations

We anticipate that the PREFER study will have a number of limitations. First, the target sample size is small, in terms of both number of study sites and number of participants, and generalizability to districts and provinces not included in the survey will require caution. Second, survey participants will provide responses about their experiences up to 6 months after ART initiation, creating the possibility of recall bias for questions pertaining to HIV testing and other pre-ART events. Third, we will largely rely on participant self-report to determine prior ART use. Several studies have demonstrated underreporting of prior ART use by self-report which could result in exposure misclassification for analyses stratified by prior treatment status. While we plan to do dried blood sampling to measure ART metabolites for prior ART exposure, the subsample who will be tested will be too small to stratify by patient or facility characteristics. Fourth, as we can only enroll participants actively participating in ART care at health facilities, the study will not capture the experiences and perceptions of individuals who have already disengaged from care. While we will be able to observe re-initiators, those who have not returned to the initiating facility will not be enrolled, potentially limiting our understanding of those who have disengaged from care. Fifth, we will utilize routinely collected EMR data to ascertain participant ART retention outcomes at 6 months after ART initiation. While efficient in terms of resources, this approach is limited to data observed at the initiating facility and will not observe participants accessing care at other facilities. Silent transfers such as these may result in outcome misclassification among some classified as disengaged.

### Ethics review

The PREFER study was approved with a separate protocol for each study country by the Boston University Institutional Review Board (South Africa H-42726, May 20, 2022; Zambia H-42903, July 11, 2022) and by the University of the Witwatersrand Human Research Ethics Committee (South Africa M220440, August 23, 2022; Zambia M210342, September 30, 2021). The protocol for South Africa was approved by Provincial Health Research Committees through the National Health Research Database for each study district (August 1, 2022 for West Rand; September 1, 2022 for King Cetshwayo and August 28, 2022 for Ehlanzeni). The protocol for Zambia was approved by the ERES-Converge IRB (2022-June-007, June 24, 2022) and the Zambia National Health Research Authority (NHRA000007/10/07/2022, July 10, 2022).

### Current status of the study

Enrollment in the PREFER survey began on September 7, 2022 in South Africa and September 21, 2022 in Zambia. We anticipate that the quantitative survey will be completed in May 2023 and all data collection by August 2023. At the time of submission of the first version of the manuscript, data collection for the quantitative survey is ongoing; data collection for the medical record review, focus group discussions, and biological samples has not yet started.

## Conclusions/discussion

Many sub-Saharan African countries have achieved the first and third of the “three 90s” ^21^—HIV testing and viral suppression among those remaining on treatment--that represented global targets for HIV programs during most of the decade of the 2010s. The second of the 90s—initiating and maintaining at least 90% of known HIV-positive people on ART—has remained out of reach for countries like South Africa and Zambia, in part due to high rates of disengagement from care after treatment initiation. For some countries, improving retention in care may be the only effective option for achieving current targets ^22^, which have been revised to the “three 95s” ^23^. To do this, attrition from care during the early treatment period—patients’ first six months on ART—must be reduced.

The PREFER study aims to understand what is happening during the early treatment period on the ground, as individual healthcare facilities interpret and implement national guidelines and healthcare workers and clients adapt to new medication regimens and new models of service delivery. PREFER was designed to understand why the early treatment period is so challenging for many patients and how service delivery can be amended to address the obstacles that lead to early disengagement from care, be they out-of-pocket costs, fear of disclosure, poor provider relations, or other concerns, and to distinguish the barriers encountered by naïve patients to those facing re-initiators.

Once the analysis has been completed, we expect to disseminate the findings from this research as broadly as possible in countries facing similar challenges with early treatment outcomes as those faced by South Africa and Zambia. Findings will be reported at project transition workshops held for local stakeholders in each country, including recipients of care, Ministries of Health, implementing partners and research organisations. Policy briefs will also be available on the project website. Within Retain6, we expect to use the information collected by PREFER to inform the second phase of the project, during which we will evaluate interventions to improve early treatment outcomes. The information collected by PREFER will also help policy makers and program managers respond to patients’ needs and design better strategies for service delivery and improve resource allocation going forward.

## Data Availability

No data are associated with this article. OpenBU. Study protocol and supplementary files for protocol manuscript in Gates Open Research. DOI: https://hdl.handle.net/2144/46276. This project contains the following data: Supplementary file 1. Screening form. Supplementary file 2. PREFER consent information sheet and signature form. Supplementary file 3. PREFER survey form. Supplementary file 4. Focus group discussion guide. Supplementary file 5. Discrete choice experiment example of choice sets. Data are available under the terms of the Creative Commons Zero "No rights reserved" data waiver (CC0 1.0 Public domain dedication).
