# Supplementary material for: Preferences for services in a patient’s first six months on antiretroviral therapy for HIV in South Africa and Zambia (PREFER): research protocol for a prospective observational cohort study
Source: Gates Open Res. 2024 Jan 9;7:119. Originally published 2023 Sep 12. [Version 2] doi: 10.12688/gatesopenres.14682.2 (PMC10858017; doi:10.12688/gatesopenres.14682.2)
Supplement: Peer review history

# Reviewer report: Reviewer response for version 2

**DOI:** 10.21956/gatesopenres.16583.r35763

**Published:** 2024-02-09

**Authors:** Kim Engler

**Affiliations:** 1 Center for Outcomes Research and Evaluation, McGill University, Montreal, Québec, Canada

I have reviewed the authors' responses to my comments and the relevant sections of the revised manuscript. The revisions appear appropriate, and I have nothing further to add.

Is the study design appropriate for the research question?

Yes

Is the rationale for, and objectives of, the study clearly described?

Yes

Are sufficient details of the methods provided to allow replication by others?

Yes

Are the datasets clearly presented in a useable and accessible format?

Not applicable

Reviewer Expertise:

HIV, patient-reported outcome measures, qualitative methods, antiretroviral therapy adherence and its barriers, stakeholder engagement, implementation research

I confirm that I have read this submission and believe that I have an appropriate level of expertise to confirm that it is of an acceptable scientific standard.

# Reviewer report: Reviewer response for version 2

**DOI:** 10.21956/gatesopenres.16583.r35762

**Published:** 2024-02-09

**Authors:** Timothy R Muwonge

**Affiliations:** 1 Infectious Diseases Institute, Makerere University, Kampala, Uganda

This is well written, all my comments have been responded to satisfactorily.

 I have no further comments to make. 

Is the study design appropriate for the research question?

Yes

Is the rationale for, and objectives of, the study clearly described?

Yes

Are sufficient details of the methods provided to allow replication by others?

Yes

Are the datasets clearly presented in a useable and accessible format?

Yes

Reviewer Expertise:

Infectious diseases, Epidemiology, clinical trials, global health, refugee health, HIV prevention, implementation science.

I confirm that I have read this submission and believe that I have an appropriate level of expertise to confirm that it is of an acceptable scientific standard.

# Reviewer report: Reviewer response for version 1

**DOI:** 10.21956/gatesopenres.16006.r35302

**Published:** 2023-11-28

**Authors:** Kim Engler

**Affiliations:** 1 Center for Outcomes Research and Evaluation, McGill University, Montreal, Québec, Canada

Thank you for the opportunity to review the research protocol manuscript submitted by Maskey and colleagues which described the PREFER study protocol. My comments are presented below, section by section.

 Title/abstract: The background’s first 2 1/2 lines could be synthesized to provide space to explain the role or significance of the differentiated service delivery models in patient retention, for those not familiar with them. The rationale and objectives are clear. A word is missing in the protocol section: “…collection of blood samples [from] self-reported naïve participants.” Also in the protocol section, perhaps the precise objective for each component of the study could be systematically provided. This will help us better understand, for instance, the significance of looking at the prevalence of ARV metabolites indicating prior ART use. Similarly, in the conclusions, should this: “to distinguish the barriers encountered by naïve patients to those facing re-initiators” be formally a part of the study objectives or rationale, instead of in the conclusions?

 Introduction: The introduction is extremely well written and logical. The study appears highly relevant and well justified. The study design seems appropriate. However, as previously suggested, I think the introduction could benefit by ending with a list of specific objectives (beyond the general overarching goals mentioned), that would better contextualize and prepare the reader for the data collection and planned analyses sections. There are hints of these interspersed throughout the text and their consolidation in one location would be helpful. Indeed, the different data collection methods and analyses could be headed by their respective objective, as a guide.

 Protocol: Some questions about study methodology could be addressed, for instance, is patient compensation for study participation provided? More could also be said about the origins and construction of the PREFER survey. How was the content developed or chosen -who was involved? Were instruments with evidence of validity used? Were patients involved in piloting or choosing the survey content?

Is the study design appropriate for the research question?

Yes

Is the rationale for, and objectives of, the study clearly described?

Yes

Are sufficient details of the methods provided to allow replication by others?

Yes

Are the datasets clearly presented in a useable and accessible format?

Not applicable

Reviewer Expertise:

HIV, patient-reported outcome measures, qualitative methods, antiretroviral therapy adherence and its barriers, stakeholder engagement, implementation research

I confirm that I have read this submission and believe that I have an appropriate level of expertise to confirm that it is of an acceptable scientific standard, however I have significant reservations, as outlined above.

# Response

**Published:** 2023-12-27

**Authors:** Sydney Rosen

**Response to Reviewer 2 of “Preferences for services in a patient’s first six months on antiretroviral therapy for HIV in South Africa and Zambia (PREFER): research protocol for a prospective observational cohort study [version 1; peer review: 2 approved with reservations]”**

**APPROVED WITH RESERVATIONS**

*Thank you for the opportunity to review the research protocol manuscript submitted by Maskew and colleagues which described the PREFER study protocol. My comments are presented below, section by section.*

*1. Title/abstract: The background’s first 2 1/2 lines could be synthesized to provide space to explain the role or significance of the differentiated service delivery models in patient retention, for those not familiar with them. The rationale and objectives are clear. A word is missing in the protocol section: “…collection of blood samples [from] self-reported naïve participants.” Also in the protocol section, perhaps the precise objective for each component of the study could be systematically provided. This will help us better understand, for instance, the significance of looking at the prevalence of ARV metabolites indicating prior ART use. Similarly, in the conclusions, should this: “to distinguish the barriers encountered by naïve patients to those facing re-initiators” be formally a part of the study objectives or rationale, instead of in the conclusions?*

**Response**: Thank you for these suggestions. We have added a brief note about the purpose of DSD models We have added the missing word—thank you for catching that error. We have added objectives for each component of the study to the abstract to the extent possible within length limits. We have also now explained the objectives of the different components in the manuscript text, in the sections in which each component is described. We also agree that the line regarding distinguish differences between naïve and re-initiating patients belongs a part of the objectives and have moved it accordingly.

*2. Introduction: The introduction is extremely well written and logical. The study appears highly relevant and well justified. The study design seems appropriate. However, as previously suggested, I think the introduction could benefit by ending with a list of specific objectives (beyond the general overarching goals mentioned), that would better contextualize and prepare the reader for the data collection and planned analyses sections. There are hints of these interspersed throughout the text and their consolidation in one location would be helpful. Indeed, the different data collection methods and analyses could be headed by their respective objective, as a guide.*

**Response**: Thank you for the positive comments about the introduction and study. We have added the following sentence to the end of the introduction:

 “The specific objectives of the study include describing the characteristics and preferences of new and re-initiating ART clients, exploring their concerns in depth through focus groups, identifying associations between client characteristics and early treatment outcomes, and assessing differences between naïve and non-naïve treatment initiators.”

 In addition, we have made the section titles more descriptive, as you suggest.

*3. Protocol: Some questions about study methodology could be addressed, for instance, is patient compensation for study participation provided? More could also be said about the origins and construction of the PREFER survey. How was the content developed or chosen -who was involved? Were instruments with evidence of validity used? Were patients involved in piloting or choosing the survey content?*

**Response**:  Thank you for these helpful suggestions, which identify previous oversights in the manuscript. We have added the following sentence about compensation (page 6):

 “Participants will be offered light refreshments during the study, and will be given a token valued at approximately US$10 to thank them for their time and participation.”

 The PREFER survey was created by the manuscript’s authors on this manuscript, based on prior experience in creating tools asking about patient experiences in seeking care. No validated tool was used, but many individual questions were drawn from our own prior studies on ART clients’ experiences, characteristics, costs, and other topics. We have now noted that the PREFER survey instrument was developed from previous AMBIT project tools and have cited a number of previous studies relevant to this topic.

# Reviewer report: Reviewer response for version 1

**DOI:** 10.21956/gatesopenres.16006.r35301

**Published:** 2023-11-01

**Authors:** Timothy R Muwonge

**Affiliations:** 1 Infectious Diseases Institute, Makerere University, Kampala, Uganda

1. **Title and Abstract**: The title and abstract should provide a clear and concise overview of the study. The title of the manuscript is informative. In the abstract, it would be helpful to briefly mention the key objectives and expected outcomes of the study.
2. **Introduction**: The manuscript effectively highlights the importance of the research question by describing the challenges of patient retention during the early phase of ART. It also emphasizes the significance of understanding patient preferences during this period.
3. **Methods**: The manuscript provides a comprehensive overview of the study methods. However, it would be beneficial to clarify the sampling strategy in more detail, including how sites and participants were selected. Additionally, it might be useful to include a flowchart or diagram to illustrate the study design and data collection process.
4. **Data Collection**: The methods section mentions that a dried blood specimen will be collected from a subsample of South African participants. It would be helpful to specify how this subsample will be selected and how the blood samples will be used in the analysis. 
**Potential Improvements**
  The research protocol could employ Focus Group Discussions (FGDs) to understand the reasons behind loss from care in the first six months after antiretroviral (ART) initiation, this comes along with quite a number of limitations of FGDS;                                       
**Proposed research questions.**
  a) Why is the early treatment period (first six months of ART initiation) challenging?
  b) How can service delivery be amended to address the obstacles that lead to early disengagement from care?
  c) What are the patient's preferences for healthcare services during the initial six months of ART treatment.?
  d) What are the barriers encountered by naïve patients to those facing re-initiators? The information collected by PREFER will help respond to patients’ needs design better strategies for service delivery and improve resource allocation going forward.
5. **Ethical Considerations**: While the manuscript mentions that the study was approved by relevant ethics committees, it would be beneficial to provide more information about the informed consent process, including how participants were informed about the study and their rights as participants.
6. **Data Management**: The manuscript briefly discusses data management but does not mention data security and privacy. It would be important to highlight the steps taken to protect the confidentiality and privacy of participants' data.
7. **Data Analysis**: The manuscript outlines the planned data analysis, including both quantitative and qualitative components. However, it would be helpful to mention specific statistical methods that will be used for data analysis.
8. **Conclusion**: The conclusion section effectively summarizes the study's objectives and potential impact on improving retention in HIV care. The manuscript could elaborate on the broader implications of the study's findings for HIV programs in sub-Saharan Africa.
9. **Tables and Figures**: It would be beneficial to include tables or figures to visually represent key information, such as the study sites, participant demographics, or the data collection process.
10. **References**: Unable to comment on the references as the journal review guidelines do not mention the type of referencing required.

Overall, the manuscript provides a well-structured and informative research protocol. Addressing the above points will enhance the clarity and completeness of the manuscript.

Is the study design appropriate for the research question?

Yes

Is the rationale for, and objectives of, the study clearly described?

Yes

Are sufficient details of the methods provided to allow replication by others?

Yes

Are the datasets clearly presented in a useable and accessible format?

Yes

Reviewer Expertise:

Infectious diseases, Epidemiology, clinical trials, global health, refugee health, HIV prevention, implementation science.

I confirm that I have read this submission and believe that I have an appropriate level of expertise to confirm that it is of an acceptable scientific standard, however I have significant reservations, as outlined above.

# Response

**Published:** 2023-12-27

**Authors:** Sydney Rosen

**Response to Reviewer 1 of “Preferences for services in a patient’s first six months on antiretroviral therapy for HIV in South Africa and Zambia (PREFER): research protocol for a prospective observational cohort study [version 1; peer review: 2 approved with reservations]”**

*APPROVED WITH RESERVATIONS*

*Title and Abstract: The title and abstract should provide a clear and concise overview of the study. The title of the manuscript is informative. In the abstract, it would be helpful to briefly mention the key objectives and expected outcomes of the study.*

**Response**: We have added to the abstract a more explicit statement of the primary objective of the study, as follows:

 “Its primary objective is to describe and understand the needs and preferences of initiating and re-initiating ART clients in order to inform the design of DSD models for the early HIV treatment period and improve early treatment outcomes.”

 Because of length constraints for the abstract, we could not describe outcomes explicitly, but we believe that the outcomes are clear from the rest of the abstract text.

*Introduction: The manuscript effectively highlights the importance of the research question by describing the challenges of patient retention during the early phase of ART. It also emphasizes the significance of understanding patient preferences during this period.*

**Response**: Thank you, we are glad that the Introduction section is clear.

*Methods:*

*1. The manuscript provides a comprehensive overview of the study methods. However, it would be beneficial to clarify the sampling strategy in more detail, including how sites and participants were selected. Additionally, it might be useful to include a flowchart or diagram to illustrate the study design and data collection process.*

**Response**: We would be glad to provide clarification where we can. The description of the selection of study sites on page 5 (and of the sites themselves in Table 1) seems complete; we do not know what other information we could add to clarify it further. In the interval since we originally submitted this manuscript, however, a paper about the SENTINEL survey, for which the PREFER sites were originally chosen, has been published and explains the selection process in a bit more detail. We now cite that paper to provide further information for readers.

 Selection of participants is described on page 6. We have tried to clarify the steps and added additional information about the consent process. We thank the Reviewer for the suggestion of a flowchart, which we have now added to the Methods section as Figure 1.

*2. Data Collection: The methods section mentions that a dried blood specimen will be collected from a subsample of South African participants. It would be helpful to specify how this subsample will be selected and how the blood samples will be used in the analysis.*

 Response: We apologize that the previous version did not clearly state how we would select the subsample. We identified 3 sites (one in each province) within our 18 participating facilities that had providers who were willing to attend training and create DBS using the venous samples they collected as part of the routine blood test performed at ART initiation. Clients enrolling at these facilities who meet the inclusion criteria for PREFER and enroll on their day of ART initiation after the necessary supplies to create DBS specimens are procured and facility staff have been trained will be enrolled consecutively. We have added language to clarify this (page 8) and to describe how we will use the data to estimate the proportion who have evidence of recent ART usage (page 9).

*3. Potential Improvements. The research protocol could employ Focus Group Discussions (FGDs) to understand the reasons behind loss from care in the first six months after antiretroviral (ART) initiation, this comes along with quite a number of limitations of FGDS;*

*Proposed research questions.*

**

*a) Why is the early treatment period (first six months of ART initiation) challenging?*

*b) How can service delivery be amended to address the obstacles that lead to early disengagement from care?*

*c) What are the patient's preferences for healthcare services during the initial six months of ART treatment.?*

*d) What are the barriers encountered by naïve patients to those facing re-initiators? The information collected by PREFER will help respond to patients’ needs design better strategies for service delivery and improve resource allocation going forward.*

**Response**: Thank you for this suggestion. The research protocol does employ FGDs, as described on page 7. Your suggested research questions are similar to those in our FGD guide, which we have now added as a supplemental file. In addition, after submitting the original version of this manuscript for review, we decided to include a brief discrete choice experiment for focus group participants. This is now described in detail in the same section of the manuscript.

*4. Ethical Considerations: While the manuscript mentions that the study was approved by relevant ethics committees, it would be beneficial to provide more information about the informed consent process, including how participants were informed about the study and their rights as participants.*

**Response**: The informed consent process is described on page 6. To provide the additional information requested, we have now added the following sentences:

 “Participants will also be informed of the risks or discomforts that may come from study participation, their rights as participants, data security and confidentiality, and information to contact the study team if questions or concerns arise.”

 “Participants will be offered light refreshments during the study and will be given a token valued at approximately US$10 to thank them for their time and participation.”

*5. Data Management: The manuscript briefly discusses data management but does not mention data security and privacy. It would be important to highlight the steps taken to protect the confidentiality and privacy of participants' data.*

**Response**: Thank you for this suggestion. We have added the following paragraph to the data management section (page 9):

 “Data for this study will be accessible only by key study personnel. Study ID numbers will be used in place of names or identifying information to safeguard participants’ privacy. All physical consent forms, questionnaires, and notes will be stored in locked cabinets, with access restricted to study personnel. All data will be collected on encrypted devices and stored on secure drives, with access restricted to study personnel. A fully anonymized data set may be posted to a public data repository when the protocol has been closed, if required by the funder and journal.”

*6. Data Analysis: The manuscript outlines the planned data analysis, including both quantitative and qualitative components. However, it would be helpful to mention specific statistical methods that will be used for data analysis.*

**Response**: Page 9 of the manuscript lays out the analysis plan, including proposed general methods. More specific statistical methods will depend to some extent on the data, and in particular our success in linking survey data and medical records for each participant. As is described in the manuscript, we expect to perform crude and adjusted log-linear regressions. Other methods may be used as deemed relevant by the study team once data have been cleaned and descriptive statistics generated. 

*7. Conclusion: The conclusion section effectively summarizes the study's objectives and potential impact on improving retention in HIV care. The manuscript could elaborate on the broader implications of the study's findings for HIV programs in sub-Saharan Africa.*

**Response**: Thank you for this suggestion. We did describe our plan to disseminate findings from this work (page 10). It is difficult to discuss the implications of the research before we know the results. To better explain the potential implications, though, we have outlined how we plan to engage local stakeholders with our findings prior to moving to phase 2 of the Retain6 project, as follows:

 “Once the analysis has been completed, we expect to disseminate the findings from this research as broadly as possible in countries facing similar challenges with early treatment outcomes as those faced by South Africa and Zambia. Findings will be reported at project transition workshops held for local stakeholders in each country, including recipients of care, Ministries of Health, implementing partners and research organizations. Policy briefs will also be available on the Retain6 website. Within Retain6, we expect to use the information collected by PREFER to inform the second phase of the project, during which we will evaluate interventions to improve early treatment outcomes. The information collected by PREFER will also help policy makers and program managers respond to patients’ needs and design better strategies for service delivery and improve resource allocation going forward.”

*8. Tables and Figures: It would be beneficial to include tables or figures to visually represent key information, such as the study sites, participant demographics, or the data collection process.*

**

**Response**: Thank you. Table 1 describes the study sites. We do not yet have information about participant demographics, as this is a protocol for prospective enrollment. We have added a figure to visually represent the data collection process (Figure 1) and hope that this will improve the clarity of our manuscript.

*9. References: Unable to comment on the references as the journal review guidelines do not mention the type of referencing required.*

**Response**: None needed

*10. Overall, the manuscript provides a well-structured and informative research protocol.*

**Response**: We thank the Reviewer for this summary.
